# Immunomodulation of T_H_2 biased immunity with mucosal administration of nanoemulsion adjuvant

**DOI:** 10.1016/j.vaccine.2016.06.043

**Published:** 2016-07-25

**Authors:** Anna U. Bielinska, Jessica J. O’Konek, Katarzyna W. Janczak, James R. Baker

**Affiliations:** a Michigan Nanotechnology Institute for Medicine and Biological Sciences, University of Michigan Medical School, Ann Arbor, MI 48109, United States; b Mary H. Weiser Food Allergy Center, University of Michigan, Ann Arbor, MI 48109, United States

**Keywords:** alum, aluminum hydroxide, cLN, cervical lymph node, ELISA, enzyme-linked immunosorbent assay, HBs, hepatitis B surface antigen, i.m., intramuscular, i.n., intranasal, i.p., intraperitoneal, NE, nanoemulsion, ova, ovalbumin, Treg, regulatory T cell, Nanoemulsion, Adjuvant, Vaccine, Immunogenicity, Intranasal vaccination

## Abstract

T_H_2-biased immune responses are associated with inadequate protection against some pathogens and with cancer, colitis, asthma and allergy. Since most currently used vaccine adjuvants induce a T_H_2-biased response, this has led to interest in developing adjuvants capable of activating T_H_1 immunity and modulating existing T_H_2 responses. Immunotherapies to shift immune responses from T_H_2 to T_H_1 have generally required prolonged immunization protocols and have not induced effective T_H_1 responses. We have demonstrated that nanoscale emulsions (NE), a novel mucosal adjuvant, induce robust IgA and IgG antibody responses and T_H_1/T_H_17 cellular immunity resulting in protection against a variety of respiratory and mucosal infections. Because intranasal (i.n.) delivery of NE adjuvant consistently induces T_H_1/T_H_17 biased responses, we hypothesized that NE could be used as a therapeutic vaccine to redirect existing T_H_2 polarized immunity towards a more balanced T_H_1/T_H_2 profile. To test this, a T_H_2 immune response was established by intramuscular immunization of mice with alum-adjuvanted hepatitis B surface antigen (HBs), followed by a single subsequent i.n. immunization with NE-HBs. These animals exhibited increased T_H_1 associated immune responses and IL-17, and decreased T_H_2 cytokines (IL-4 and IL-5) and IgG1. NE immunization induced regulatory T cells and IL-10, and IL-10 was required for the suppression of T_H_2 immunity. These data demonstrate that NE-based vaccines can modulate existing T_H_2 immune responses to promote T_H_1/T_H_17 immunity and suggest the potential therapeutic use of NE vaccines for diseases associated with T_H_2 immunity.

## Highlights

• Nanoemulsion vaccine adjuvant induces robust antigen specific T_H_1-biased immunity.
• Nanoemulsion vaccine adjuvant suppresses established T_H_2 immunity.
• Efficacy of nanoemulsion vaccine in mice with pre-existing immunity to same antigen.
• Nanoemulsion vaccines induce IL-10 and regulatory T cells.

## 1 Introduction

CD4^+^ effector T cell responses are classified according to their cytokine and transcription factor profiles, with T_H_1 and T_H_2 cells being the most widely studied types [1]. Differentiation of T_H_1 cells is driven by IL-12 secreted by macrophages and IFN-γ from T cells or NK cells, and results in the production of T_H_1-type cytokines including IFN-γ, IL-2 and TNF-α. In mice, IFN-γ enhances immunoglobulin class switching to increase production of IgG2a and IgG2b subclasses as well as activation of other T_H_1 cell-mediated effector responses [2]. Alternatively, T_H_2 responses can be initiated by IL-4-dependent differentiation of T_H_2 effector CD4^+^ cells that produce T_H_2-type cytokines, including IL-4, IL-5, IL-9 and IL-13, which can culminate in the increased production of IgG1 subclass and IgE antibodies. The T_H_1/T_H_2 paradigm is useful for classification of immune responses and becomes better defined as mechanisms of action of CD4^+^ effector T cells are further elucidated.

The type of cell-mediated immunity affects the induction of specific protective immunity to infectious diseases, inflammatory responses, allergy or autoimmunity and even can increase susceptibility to certain infections [3], [4]. This is of particular importance in vaccine development because adjuvants are able to skew helper T cell profiles, and choosing the appropriate adjuvant may influence efficacy [5]. The most widely used adjuvant alum induces strong T_H_2-associated immune responses which are less effective against pathogens for which T_H_1 cell-mediated immunity is required for clearance [6], [7], [8]. Because of this, a number of vaccines based on new T_H_1 polarizing adjuvants including liposomes, CpG-containing oligodinucleotides, monophosphoryl lipid A, and QS-21 are being evaluated both in animal studies and in clinical trials [9], [10], [11], [12]. Many of these adjuvants are under development for production of vaccines that may be used in people who have already been exposed to the same antigen or pathogen, either through prior vaccination or infection. In the case of individuals previously primed to have a T_H_2 skewed immune response from an alum-adjuvanted vaccine, it is unclear if boosting with a T_H_1-polarizing vaccine adjuvant would redirect the immune response towards a T_H_1 response, or if it would simply boost the T_H_2 responses for which the immune system had already been primed. Additionally, a considerable interest has been directed towards development of strategies for modulation of existing T_H_2 immune responses, especially for the alleviation of T_H_2-biased allergic responses [13], [14]. Adjuvants capable of redirecting established antigen-specific T_H_2 responses to induce T_H_1 while suppressing T_H_2 immunity have the potential for impacting a variety of diseases driven by aberrant T_H_2 immune responses.

Our group has developed a nanoscale oil-in-water emulsion (nanoemulsion, NE) vaccine adjuvant platform that when delivered intranasally (i.n.) induces robust systemic and mucosal responses without local inflammatory effects [15], [16], [17], [18], [19], [20]. In animal studies, i.n. immunizations with NE mixed with a variety of viral and bacteria-derived antigens, including influenza, hepatitis B, respiratory syncytial virus, vaccinia and anthrax, yields high protective antibody titers. In contrast to adjuvants like alum that induce T_H_2-biased immune responses [6], [21], [22], nasally administered NE vaccines result in T_H_1 and T_H_17 polarized immune responses [18], [23], [24]. Regardless of the model tested, T_H_2 cytokine responses in animals immunized with NE are always low, and no significant production of IgE has been observed. This is true even in BALB/c mice that are inherently biased towards a T_H_2-type response [25]. Because this NE adjuvant is a robust T_H_1-polarizing adjuvant, we hypothesized that it would be a good candidate for redirecting established T_H_2 immune responses to a more balanced T_H_1/T_H_2/T_H_17. In proof-of-concept studies presented here, we have investigated the effect of nasal administration of a NE vaccine in mice previously vaccinated with an alum-adjuvanted vaccine.

## 2 Material and methods

### 2.1 Antigen and adjuvants

The recombinant hepatitis B surface antigen (HBs) was supplied by Human Biologicals Institute (Indian Immunologics, Ltd, Hyderabad, India). The endotoxin level was determined to be <7.5 EU/20 μg of HBs, which is significantly below the internationally accepted standard of ⩽30 EU/20 μg of protein. Ovalbumin (ova) was purchased from Sigma–Aldrich. Ova peptides, class I-restricted ova 257–264 (SIINFEKL, ova I) and class II-restricted ova 323–339 (ova II) were purchased from Invitrogen. Nanoemulsion adjuvant (NE) was supplied by NanoBio Corporation, Ann Arbor, MI. NE was produced by a high speed emulsification of ultra pure soybean oil with cetyl pyridinium chloride, Tween 80 and ethanol in water, with resultant NE droplets with average 350–400 nm diameter [18]. Aluminum hydroxide (alum) was purchased from Sigma–Aldrich, Inc. All reagents were tested for the presence of endotoxin using RAW-Blue cell-based assay *in vitro* (InvivoGen, San Diego, CA).

### 2.2 Mice and immunizations

**Fig. 1:**
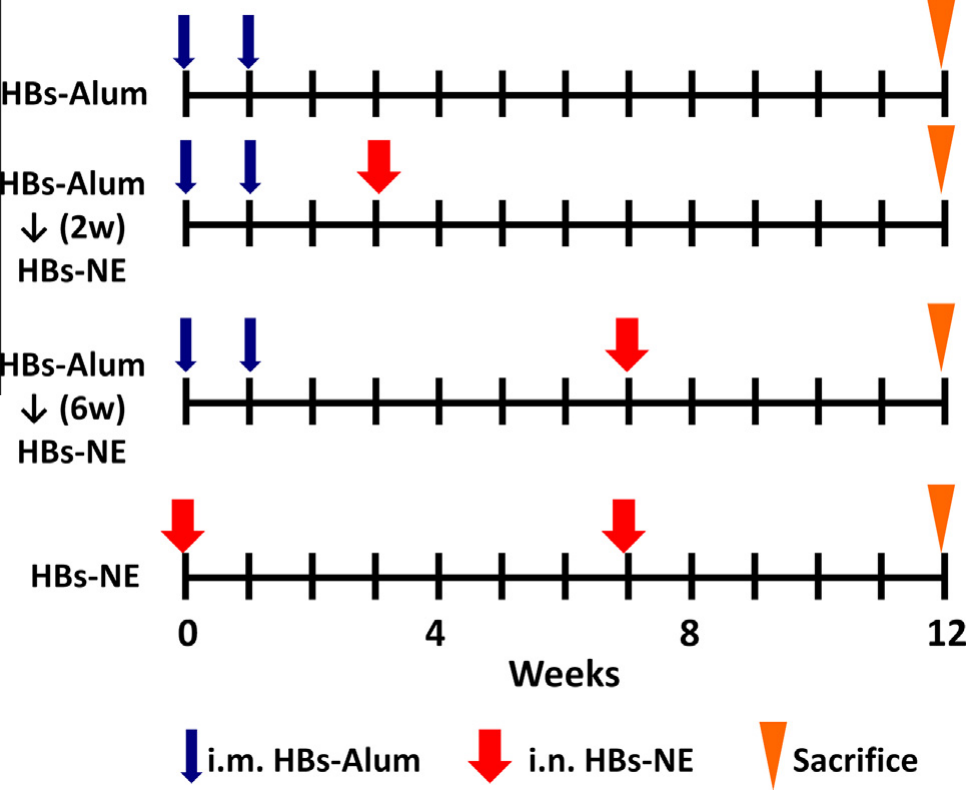
Design and schedule of immunomodulation studies.

Pathogen-free CD-1 mice (females 6–8 weeks old) were purchased from the Charles River Laboratories. All animal procedures were performed according to the University Committee on the Use and Care of Animals at the University of Michigan. Immunization schedule is shown in Fig. 1. For all immunizations, mice were anesthetized under isoflurane anesthesia using the IMPAC6 precision vaporizer. Intranasal (i.n.) immunizations were done using a pipette tip by administration of 5 μl/nare of formulation containing 20 μg of antigen mixed with 20% NE. Antigen mixed with PBS alone served as a control. Intramuscular immunizations (i.m.) were performed by injection of 50 μl containing 20 μg of antigen adsorbed on 0.5 mg/ml alum into the epaxial muscle as described previously [18]. Sera were obtained by saphenous vein bleeding, and splenocytes were harvested at the end of the experiment. In IL-10 depletion experiments, mice were injected i.p. with 1 mg anti-IL-10 (purified from rabbit serum [26]) or control rabbit IgG 12 h before and 2 days after NE immunization.

### 2.3 Measurement of serum IgG subclasses

Serum antibody and IgG subclasses titers were determined by ELISA, with plates coated with 5 μg/ml of HBs as described previously [18].

### 2.4 Analysis of cytokine expression

Single cell suspensions of freshly isolated mouse splenocytes were cultured at 4 × 10^6^ cells/ml with or without antigen (10 μg/ml). After 48 h, supernatants were collected and analyzed for the presence of cytokines using Milliplex Mouse Cytokine/Chemokine Immunoassay Kit (Millipore, Billerica, MA).

### 2.5 Measurement of the induction of regulatory T cells (Tregs) after NE immunization

Mice were immunized i.n. with ova and NE (ova-NE) or non-adjuvated ova (ova-PBS) at weeks 0 and 4. Splenocytes were harvested at 1 and 7 weeks after the first immunization. Red blood cell depleted single cell suspensions were stained by flow cytometry to quantify regulatory T cells. Fc receptors were blocked with purified anti-CD16/32 (clone 93, BioLegend) and surface markers were stained using antibodies against CD3 (145-2C11), CD4 (RM4-5) and CD25 (7D4) (all from eBioscience or BD Biosciences), permeabilized, fixed and labeled for intracellular Foxp3 (FJK-16s). Samples were acquired on an Accuri C6 flow cytometer (BD Biosciences). Data were analyzed using FlowJo (Treestar).

### 2.6 Statistics

Results are presented as the geometric mean ± 95% confidence interval. Statistical comparisons were assessed by the Mann–Whitney test using GraphPad Prism version 6 (GraphPad Software). The *p* value < 0.05 was considered as significant. In every reported result the data shown are representative of at least 2 experiments.

## 3 Results

### 3.1 Mucosal immunization with NE adjuvant modifies T_H_2 polarized immune response

**Fig. 2:**
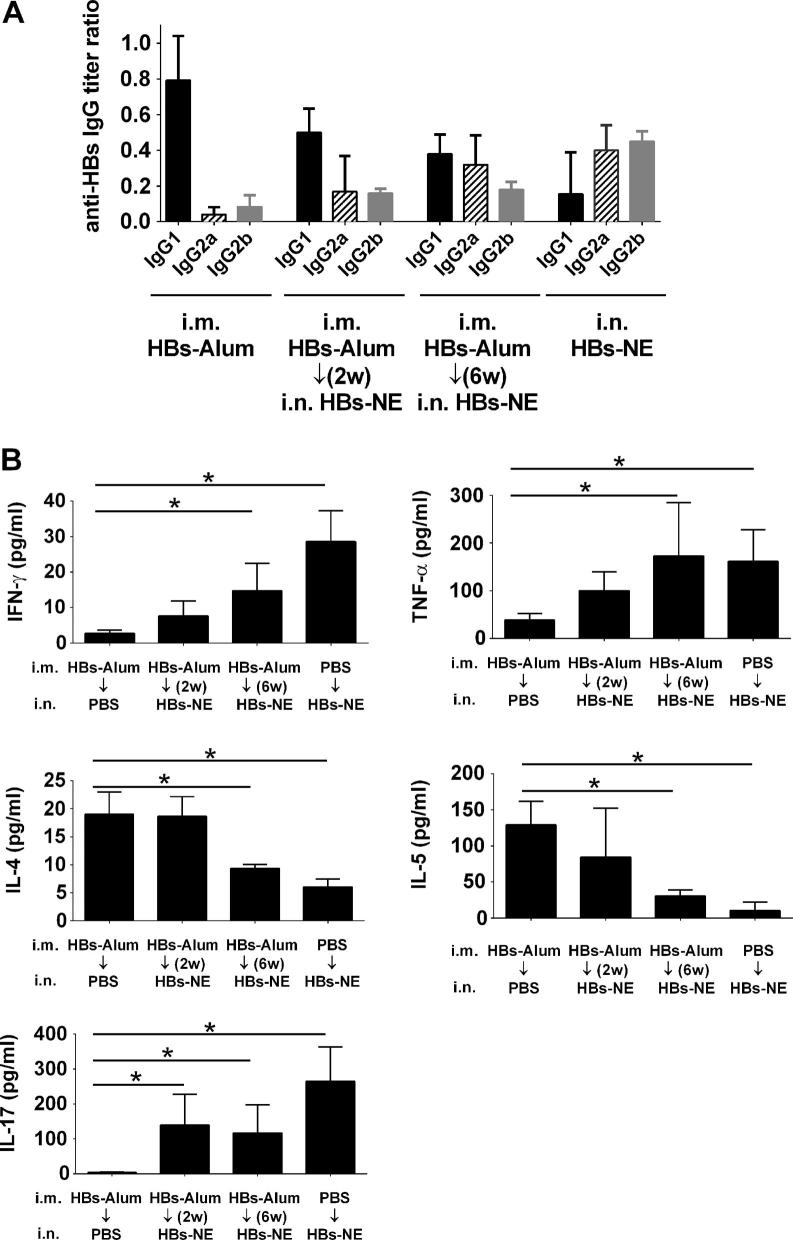
Modulation of T_H_2 immunity with NE adjuvant. Mice were immunized i.m. with HBs-alum to induce T_H_2 immunity. Mice were subsequently immunized i.n. with HBs-NE. (A) Serum HBs-specific antibody subclass titers determined at week 12 are expressed as ratios of the endpoint titer of each subclass with total IgG titer. (B) Cellular recall immune responses to HBs protein were measured in splenic lymphocytes stimulated *ex vivo* with 5 μg/ml HBs for 48 h. Cytokine secretion has been normalized to control unstimulated splenocyte cultures. Data are expressed as mean ± standard deviation (*n* = 5). Statistically significant differences (*p* < 0.05) are indicated by ^∗^.

To elicit the T_H_2 response, mice were immunized with two i.m. injections of 20 μg HBs adsorbed on alum [21]. Analysis of serum IgG subclass and cytokine expression confirmed that HBs-alum immunization yielded predominantly IgG1 antibody subclass (Fig. 2A) and induction of T_H_2-type cytokines IL-4 and IL-5 (Fig. 2B). There was no change in antibody titers in mice receiving only the HBs-alum vaccine from weeks 6–12 (data not shown). To investigate whether NE adjuvant can modify this T_H_2 bias, the mice were subsequently immunized with a single intranasal administration of HBs-NE at 2 or 6 weeks after the second HBs-alum sensitization (Fig. 1). Serum IgG analysis showed significant increases in IgG2a and IgG2b subclasses following HBs-NE immunization, with antibody titers comparable to the HBs-NE immunization in mice that did not receive the HBs-alum vaccine (Fig. 2A). Antigen-specific cytokine expression in splenic lymphocytes after the 6 week NE immunization showed significant induction of T_H_1-type IFN-γ and TNF-α and the T_H_17 cytokine IL-17(Fig. 2B) and decreased IL-4 and IL-5 production in mice immunized with HBs-NE six weeks after HBs-alum sensitization. This effect was not significant in mice immunized with HBs-NE at an earlier time point (2 weeks). Nasal immunization with HBs-NE alone was used as a control to assess modulation of established T_H_2 immunity with NE adjuvant, and antibody and cytokine patterns were similar after HBs-NE immunization regardless of whether the mice had been previously T_H_2 sensitized or not (Fig. 2A and B). There was a slight decrease in IFN-γ and IL-17 in mice that received both vaccines compared with mice immunized with NE only, however these differences were not statistically significant (*p* = 0.70 and 0.41, respectively).

**Fig. 3:**
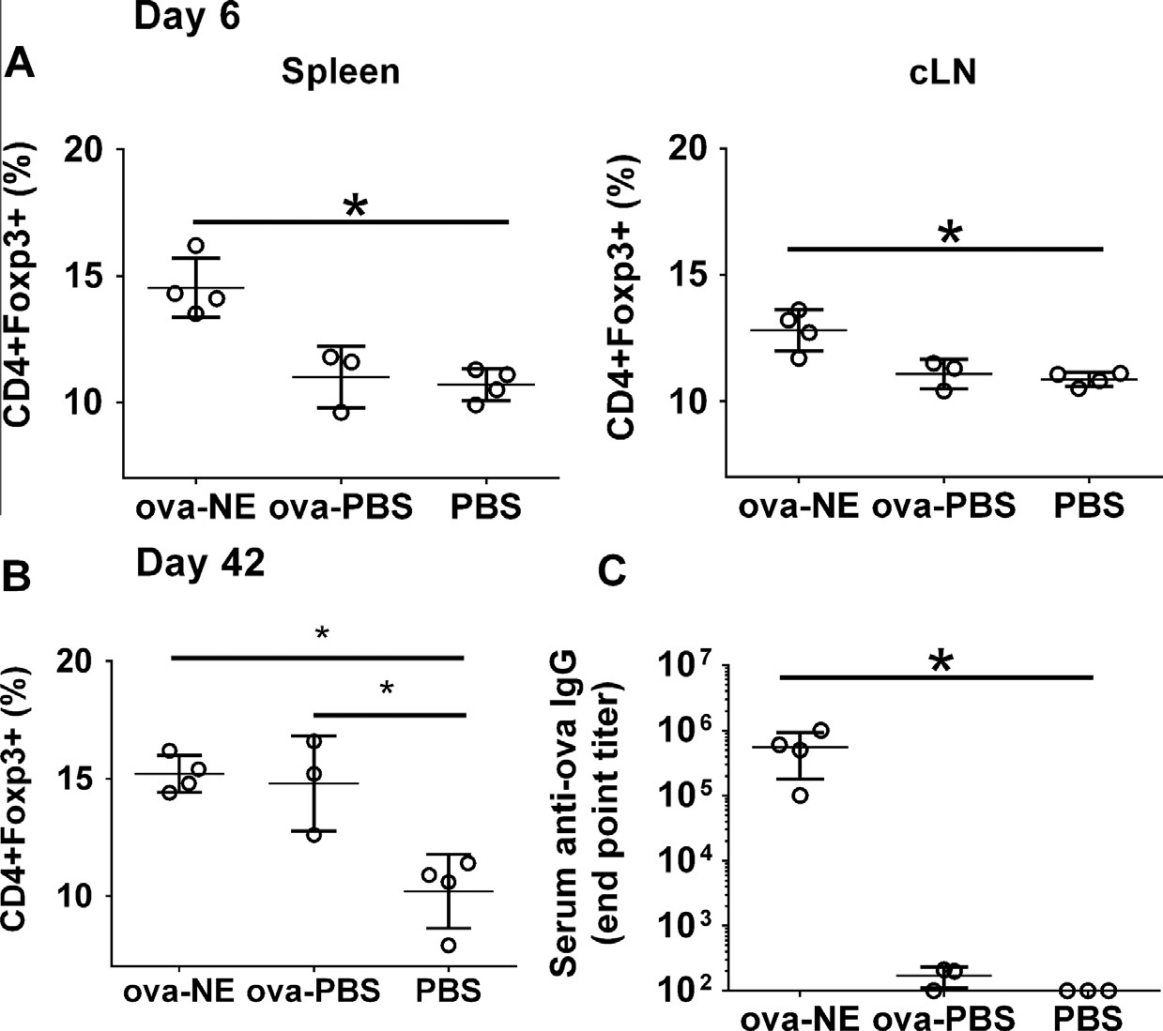
Nasal immunization with NE adjuvant increases frequency of CD4^+^FoxP3^+^ regulatory T cells. Mice were immunized i.n. with ova-NE (*n* = 4) or ova-PBS (*n* = 3). Splenic and cervical lymph node cells (cLN) were isolated from the mice and stained with CD4, CD25 and Foxp3 at (A) 6 and (B) 42 days after immunization. (C) Serum ova-specific IgG induction was determined at day 42. Statistically significant differences (*p* < 0.05) are indicated by ^∗^.

To investigate a potential role of regulatory T cells (Tregs) in the mechanism of NE adjuvant, mice were immunized i.n. with ova-NE or with non-adjuvanted ova in PBS (ova-PBS) as a control. Treg frequency (CD4^+^ Foxp3^+^) was measured after 6 days both in the nasal draining lymph nodes (cervical lymph nodes, cLN) and in the periphery in splenic lymphocytes. Analysis of CD4^+^Foxp3^+^ T cells showed that mucosal administration of ova-NE induced significantly more Treg expansion in both cLN and spleen compared to ova-PBS and PBS administration (*p* < 0.03) (Fig. 3A). Interestingly, by 6 weeks after immunization the frequency of Tregs was elevated in both ova-NE and ova-PBS groups (Fig. 3B). Consistent with results documented previously, the i.n. immunization with ova-NE induced a potent IgG response, while no significant titers were detected in ova-PBS immunized mice (Fig. 3C).

**Fig. 4:**
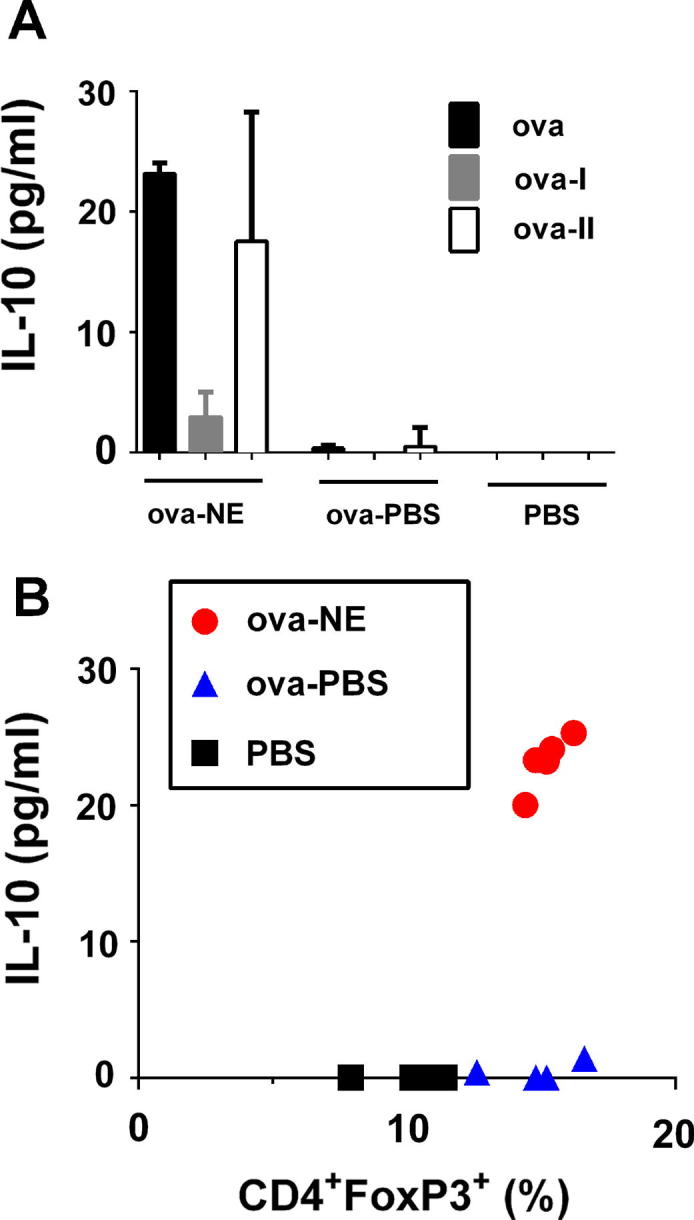
Correlation of Treg frequency and antigen specific IL-10 expression. Mice (*n* = 5) were immunized i.n. with ova-NE or ova-PBS. 42 days after immunization, splenocytes were harvested and stimulated with ova, or ova-I or ova-II peptides for 48 h. (A) IL-10 secretion in cell culture determined by Milliplex. (B) Splenic Treg frequency and production of IL-10 after stimulation with ova were plotted for each individual mouse to demonstrate the correlation between Tregs and IL-10 expression for NE immunized mice.

Further analysis revealed that production of IL-10, a suppressive cytokine associated with Treg function [27], [28], was increased in cells from mice immunized with i.n. ova-NE (Fig. 4A). Furthermore, IL-10 production was only significantly induced with stimulation with ova protein or a MHC II-restricted ova peptide, not a MHC I ova peptide, suggesting that the IL-10 is produced by CD4^+^ T cells. Correlates of Treg frequency vs. IL-10 expression show no IL-10 production in mice immunized with ova-PBS despite the increase in frequency of Treg frequency. In contrast, in mice treated with ova-NE there was a significant increase in IL-10 levels that closely correlated with increased Treg frequency (Fig. 4B).

**Fig. 5:**
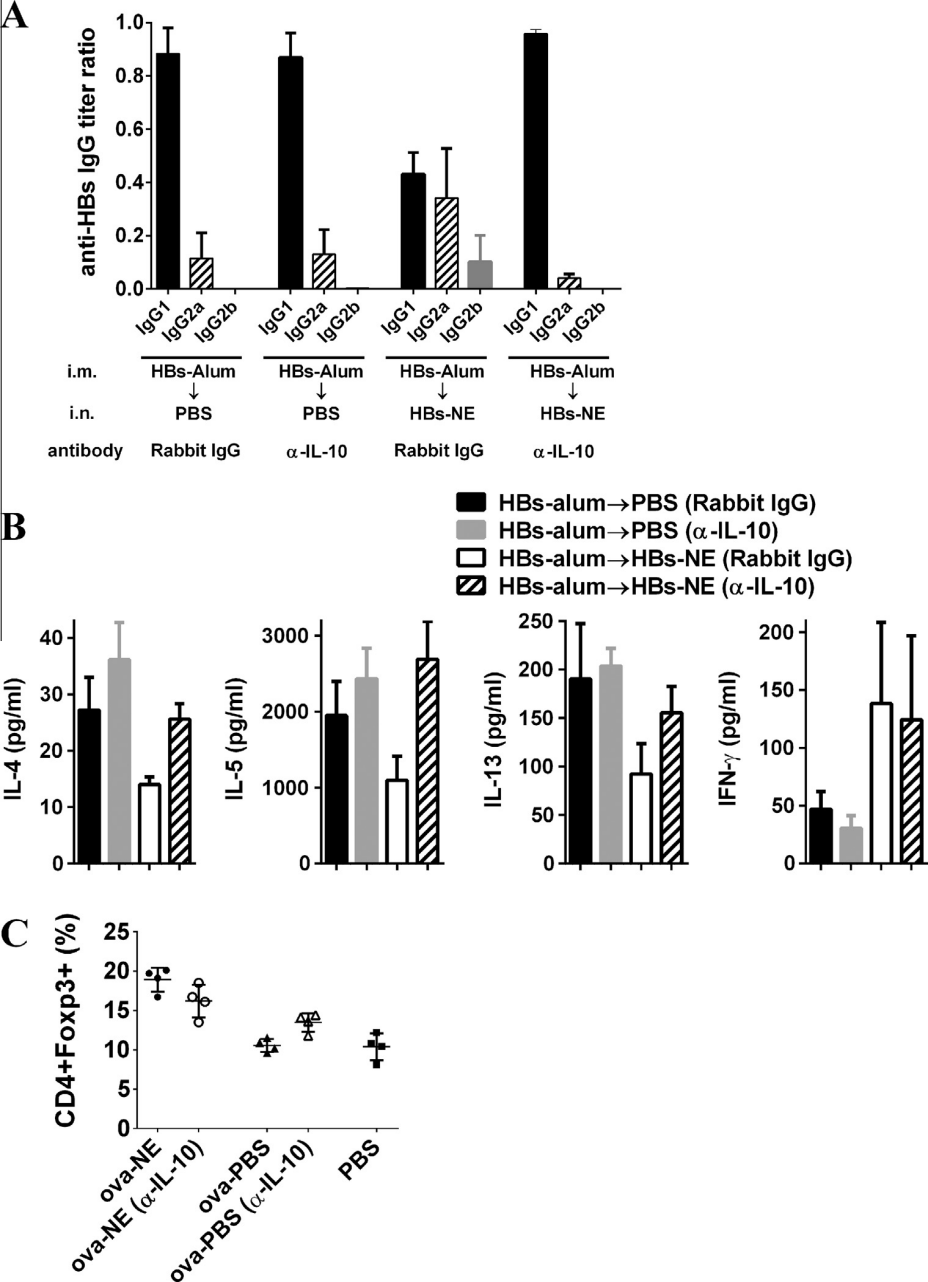
Suppression of TH2 immunity by NE adjuvant requires IL-10. (A and B) Mice (*n* = 5) were immunized i.m. with HBs-alum at weeks 0 and 1 followed by i.n. HBs-NE at week 7. Anti-IL-10 antibodies or rabbit IgG control were administered at the time of NE immunization. (A) Serum HBs-specific antibody subclass titers determined at week 12 are expressed as ratios of the endpoint titer of each subclass with total IgG titer. (B) Cellular recall immune responses to HBs protein were measured in splenic lymphocytes stimulated *ex vivo* with 5 μg/ml HBs for 48 h. Cytokine secretion has been normalized to control unstimulated splenocyte cultures. Data are expressed as mean ± standard deviation. (C) Mice were immunized i.n. with ova-NE or ova-PBS. Cells from cLN were isolated from the mice and stained with CD4, CD25 and Foxp3 at 6 days to measure Treg frequency.

In order to determine the effects of IL-10 on NE-mediated suppression of the alum-induced T_H_2 immune response, mice were immunized i.m. with HBs-alum and IL-10 was depleted at the time of HBs-NE immunization. There was no balancing of IgG subclasses when IL-10 was depleted during NE immunization (Fig 5A), and the subclass pattern was similar to that observed from mice only receiving the HBs-alum vaccine. The suppression of T_H_2 cytokines (IL-4, IL-5, IL-13) did not occur upon HBs-NE immunization with simultaneous IL-10 depletion. The induction of the T_H_1 cytokine, IFN-γ, was not inhibited by IL-10 depletion. IL-10 depletion did not significantly change the percentage of Tregs induced by NE immunization.

## 4 Discussion

The development of new materials and adjuvants that can modulate the immune system is an emerging field in immunology, with interests in multiple settings, including vaccine development and allergy [13], [29], [30], [31]. In this proof of concept study we present a new adjuvant-based approach to immunomodulation in mice. We have demonstrated that immunization with novel oil-in-water nanoemulsion adjuvant not only produced robust cellular and humoral immunity but also redirected existing T_H_2-biased responses towards a more balanced T_H_1/T_H_2 phenotype in a model of established antigen-specific T_H_2 immunity.

In contrast to the commonly used aluminum adjuvant(s), NE is not associated with the T_H_2 phenotype. Consistent with our previous results [16], [17], [18], immunization with NE adjuvant produced T_H_1 biased immunity with IFN-γ and TNF-α production (Fig. 2B). The significant increase of IgG2a and IgG2b antibodies and T_H_1 type cytokines and simultaneous reduction of IgG1 antibodies and T_H_2 cytokines demonstrates that NE adjuvant is capable of shifting an established T_H_2 response towards a more balanced cell-mediated immunity both through the induction of T_H_1 and suppression of T_H_2. In mice, IgG1 is regulated via a T_H_2/IL-4 pathway, and in numerous studies IgG1 has been used as a robust indicator for the assessment of a T_H_2 response [32], [33]. Mucosal HBs-NE immunization of HBs-alum sensitized mice diminished IgG1/IgG2a and IgG1/IgG2b ratios from 10.46 and 8.67, to 1.2 and 2.1, respectively, clearly demonstrating modulation of the HBs-specific immune response.

Analysis of cytokine expression provided direct assessment of T cell activation. Elevated IFN-γ and diminished IL-4 levels after antigen stimulation of splenic lymphocytes indicated that HBs-NE immunization resulted in T_H_2 cell suppression and a shift to T_H_1 response (Figs. 2B and 3B). This effect was not detected in splenocytes from mice immunized with NE at an earlier time point (2 weeks), despite increase in IgG2a and IgG2b antibodies in comparison to HBs-alum controls (Fig. 2). This result indicates that while NE has the potential to modify established T_H_2 immunity, effective modification of an ongoing immune response may depend on the schedule and number of immunizations.

Intranasal immunization with NE adjuvant induces a T_H_17 immune response [23]. The antigen-specific IL-17 expression was also detected in splenocytes of mice with T_H_1 redirected immune response (Fig. 2B). Despite association with various autoimmune disorders, T_H_17 also contributes to host defense as a T cell subset involved in protection against extracellular pathogens [34] and has been shown to play a critical role in the efficacy of several vaccines [35], [36], [37], [38], [39]. Although excessive prolonged IL-17 production may contribute to pathophysiology of respiratory infections or asthma and allergy, the degree of T_H_17 induction with NE immunization is much lower than levels typically observed in diseases in which IL-17 contributes to pathology [40], [41]. The effect of NE-induced IL-17 production on T_H_2/T_H_1 immunomodulation remains to be investigated; however, T_H_17 cell-mediated immunity may suppress IgE responses, as has been recently indicated for T_H_17 immunity associated with human autoimmune disease [42].

The exact mechanism of action of NE adjuvant is not yet fully elucidated. NE is formulated using ultrapure and endotoxin-free components and does not contain any commonly recognized TLR agonists or ligands [18]. However, our recent results demonstrate involvement of the TLR pathway in immunogenicity of NE adjuvant both *in vivo* and *in vitro* [24]. NE facilitates antigen uptake and trafficking into lymphoid tissue while not causing either nasal irritation or disruption of mucosal epithelial architecture [43], [44]. NE-mediated enhancement of antigen internalization and processing by the antigen presenting cells could be important for the optimal antigen presentation to T cells and development of T_H_1 biased immunity [45], [46], [47]. We have shown that intranasal treatment with NE adjuvant does not produce significant amounts of IFN-γ, TNF-α, IL-12, IL-4, IL-5, IL-9, IL-13 or inflammatory cytokines such as IL-1β in the nasal mucosa [43]. Based on the absence of inflammatory mediators, rhinitis or cellular infiltrates at the high 20% concentration, NE appears to be non-inflammatory and is generally biocompatible with mucosal and pulmonary tissue in mice, rats, guinea pigs, dogs and humans (not shown and [18], [44], [48]).

While data from mouse models clearly show that alum drives T_H_2 immunity, the evidence for T_H_2 skewing by alum based vaccines in humans is not entirely clear. A few clinical studies have shown that alum induces a mixed T_H_2 and T_H_1 response, but the overall effect across various antigens in humans as compared to mice is poorly defined [49], [50], [51], [52]. Additionally, studies assessing immune polarization induced by alum mainly have been performed in adults. Given that neonatal immune systems are inherently biased towards T_H_2 [53], [54], the immunization of newborns with an alum-based Hepatitis B vaccine raises concerns about the role vaccines might play in the growing issue of allergic disease in young people [55], [56]. Moving forward, it may be advantageous to consider vaccine adjuvants that induce required protective immunity without activating T_H_2 polarized responses. While the ability of NE adjuvant to shift towards T_H_1 in humans is not explored in this study, in a Phase I clinical trial a flu vaccine containing this NE adjuvant formulation induced T_H_1 antigen-specific IgG, neutralizing antibody, as well as mucosal IgA, demonstrating the immunogenicity of this adjuvant in humans [44].

Here, we demonstrate that NE immunization resulted in the induction of Tregs in both the draining lymph nodes and the periphery. The correlating increase in IL-10, suggests that these Tregs may have suppressive function, and likely play a role in the immune responses induced by NE [57]. Tregs are considered essential for the maintenance of immunological homeostasis and for the control of exacerbated immune responses. Numerous studies have demonstrated a role for Tregs in restraining exacerbated immune responses during natural infection, suggesting that Treg depletion and/or inactivation could improve efficacy of vaccines [58], [59]. Much less is known regarding the role of Tregs in the induction and maintenance of protective immune response with various adjuvant-based vaccines; however the data presented here suggest that for NE the induction of Tregs does not inhibit overall vaccine efficacy but may be responsible for the suppression of the T_H_2 response. It has previously been reported that antigen-specific T_H_1 and regulatory T cells can mediate modification of IgG subclass pattern [60], consistent with the data presented here. Since Tregs induced with antigen alone are often considered as immune suppressors in the process of immune tolerance, our results may suggest a functional difference between the Treg populations in mice immunized with antigen alone compared with antigen and NE. Similarly, these results may suggest a functional difference between Treg populations generated in various modes of i.n. immunization. Further characterization of Treg function and direct functional assessment of their suppressive potential will help to clarify their role in NE induced immune response.

IL-10 production is one mechanism by which immune responses can be suppressed. Not only does NE induce IL-10, but depletion of IL-10 during NE immunization alters the ability of NE to suppress T_H_2 immunity (Fig. 5). Interestingly, IL-10 depletion did not alter T_H_1 induction by NE, so IL-10 does not appear to be involved in the induction of immune responses by NE. IL-10 was depleted during immunization but not at the time of sacrifice when the recall response to antigen was determined, suggesting that IL-10 is critical for priming of cellular immune responses that result in a shift from T_H_2 to T_H_1 in this model.

## 5 Conclusions

Our initial results suggest the usefulness of NE-based delivery systems in the development of therapeutic vaccines to modify T_H_2 immune responses, as well as the ability of NE-based vaccines to retain their immune phenotype even in individuals that received previous vaccinations with the same antigen and other adjuvants. This novel approach to immunomodulation using i.n. delivery of NE adjuvant to produce mucosal immunity and a systemic T_H_1-biased immune response could be useful for the development of vaccines to induce antigen-specific T_H_1 immune responses even in individuals with pre-existing T_H_2 biased immunity. This suggests that NE adjuvant may be especially useful in situations where pathologies are due to aberrant T_H_2 immune response, such as allergy.

## Grant support

This project has been funded by the Bill and Melinda Gates Foundation, under award 37868 and the National Institute for Allergy and Infectious Disease, National Institutes of Health under Contract No. HHSN272200900031C.
